# Electroencephalogram–Electromyography Coupling Analysis in Stroke Based on Symbolic Transfer Entropy

**DOI:** 10.3389/fneur.2017.00716

**Published:** 2018-01-04

**Authors:** Yunyuan Gao, Leilei Ren, Rihui Li, Yingchun Zhang

**Affiliations:** ^1^College of Automation, Intelligent Control & Robotics Institute, Hangzhou Dianzi University, Hangzhou, China; ^2^Department of Biomedical Engineering, University of Houston, Houston, TX, United States; ^3^Guangdong Provincial Work-Injury Rehabilitation Hospital, Guangzhou, China

**Keywords:** corticomuscular coupling, symbolic transfer entropy, stroke, electroencephalogram, electromyography

## Abstract

The coupling strength between electroencephalogram (EEG) and electromyography (EMG) signals during motion control reflects the interaction between the cerebral motor cortex and muscles. Therefore, neuromuscular coupling characterization is instructive in assessing motor function. In this study, to overcome the limitation of losing the characteristics of signals in conventional time series symbolization methods, a variable scale symbolic transfer entropy (VS-STE) analysis approach was proposed for corticomuscular coupling evaluation. Post-stroke patients (*n* = 5) and healthy volunteers (*n* = 7) were recruited and participated in various tasks (left and right hand gripping, elbow bending). The proposed VS-STE was employed to evaluate the corticomuscular coupling strength between the EEG signal measured from the motor cortex and EMG signal measured from the upper limb in both the time-domain and frequency-domain. Results showed a greater strength of the bi-directional (EEG-to-EMG and EMG-to-EEG) VS-STE in post-stroke patients compared to healthy controls. In addition, the strongest EEG–EMG coupling strength was observed in the beta frequency band (15–35 Hz) during the upper limb movement. The predefined coupling strength of EMG-to-EEG in the affected side of the patient was larger than that of EEG-to-EMG. In conclusion, the results suggested that the corticomuscular coupling is bi-directional, and the proposed VS-STE can be used to quantitatively characterize the non-linear synchronization characteristics and information interaction between the primary motor cortex and muscles.

## Introduction

Electroencephalogram (EEG) is a non-invasive brain imaging technique that uses scalp electrodes to measure the voltage fluctuations induced by the mass electrical activity of neurons (1). Electromyography (EMG) technique is usually used to record the electrical activity produced by skeletal muscles (2). In the process of movement, the central nervous system associated with relevant brain regions and the peripheral nerve system associated with specific muscles is automatically synchronized in addition to the synergistic effect between different brain regions (1, 2). As such, the synchronization strength reflects the interaction between the primary motor cortex and the muscles and provides theoretical basis for the rehabilitation of stroke and dyskinesia patients (3).

Since Conway et al. (4) first reported a correlation between EEG and EMG in the process of exercise in 1995, dynamic interactions between brain activities and muscle motion feedbacks have been widely investigated. It was found that the coherence of EEG–EMG signals is closely related to the motion tasks (5). For instance, the oscillation in the beta band is associated with mild-to-moderate isometric contraction, and the oscillation in the low range of the gamma band is related to strategies for controlling stronger muscle force production and dynamic movements (6). Various coherence analysis techniques, including cortical–muscular functional coupling (7, 8), Granger causality analysis (9, 10), transfer entropy (TE) analysis (11, 12), and symbolic transfer entropy (STE) analysis (13) have been developed and applied to EEG and EMG signal coupling analysis. Among these, the STE technique is an effective method to analyze the relationship between neural and muscular activity coupling. In general, the STE yields characteristics not depending on the established model and non-linear quantitative analysis (14). It can be used to estimate the functional coupling strength and information transfer direction between cortices and muscles and to reveal movement control and response mechanisms during movements (15). For instance, the STE has been used to analyze the non-linear functional connection between EEG single and surface EMG signals of hand muscles (16), which demonstrated that the functional corticomuscular coupling is significant in the beta band in the static force output for healthy subjects.

However, the STE also holds ineligible challenges in practice. For example, the number of symbols applied in the time sequence in traditional STE is fixed, which is therefore not flexible and dynamic characteristics of signals are easily lost. In addition, the STE has only been applied to health subjects so far, has not been tested in stroke patient population yet (16–18).

To bridge this gap, a variable scale symbolic transfer entropy (VS-STE) analysis approach was developed in this study to better investigate the corticomuscular coupling in both post-stroke patients and healthy volunteers. In particular, the corticomuscular coupling strength was assessed based on the EEG signals measured from the motor cortex and EMG signals obtained from upper limb in both time-domain and frequency-domain. The EEG–EMG coupling strength of subjects were also quantitatively evaluated in terms of significant area, which provided evidence to apply corticomuscular coupling in the rehabilitative evaluation of motor function disorders.

## Materials and Methods

### Experimental Design

#### Participants

Twelve male subjects, including a control group (*n* = 7, age: 25.7 ± 1.11 years, all right handed) and a patient group (*n* = 5, age: 47.8 ± 2.28 years) were recruited in this study. The details of the subjects are summarized in Table 1, where S1, S2, and S3 are patients with mild stroke, S4 and S5 are patients with severe stroke, and S6–S12 are healthy volunteers. The study protocol was approved by the Institutional Review Board of Guangdong Provincial Work Injury Rehabilitation Hospital. Prior to the experiment, all the subjects were informed of the details of the experiments and signed the informed consent form.

**Table 1:** Demographic information the subjects.

Subject #	Age	Used hand	Status	Condition
S1	45	Left	Suffering from stroke for 2 months	Small amount of bleeding in right intracranial brain, left foot cannot walk flexibly
S2	47	Right	Suffering from stroke for 1 month	Right brain intracranial hemorrhage, limbs can only complete the basic actions
S3	49	Right	Suffering from stroke for 1 month	Right brain intracranial hemorrhage, limbs can complete basic movements
S4	51	Right	Suffering from stroke for 2 months	Right brain intracranial hemorrhage, upper limbs can only complete simple actions
S5	47	Right	Suffering from stroke for 1 month	Right brain intracranial hemorrhage, upper limbs can only complete simple actions
S6	27	Right	Healthy	No
S7	26	Right	Healthy	No
S8	24	Right	Healthy	No
S9	25	Right	Healthy	No
S10	27	Right	Healthy	No
S11	26	Right	Healthy	No
S12	25	Right	Healthy	No

#### Experimental Paradigm

**Figure 1:**
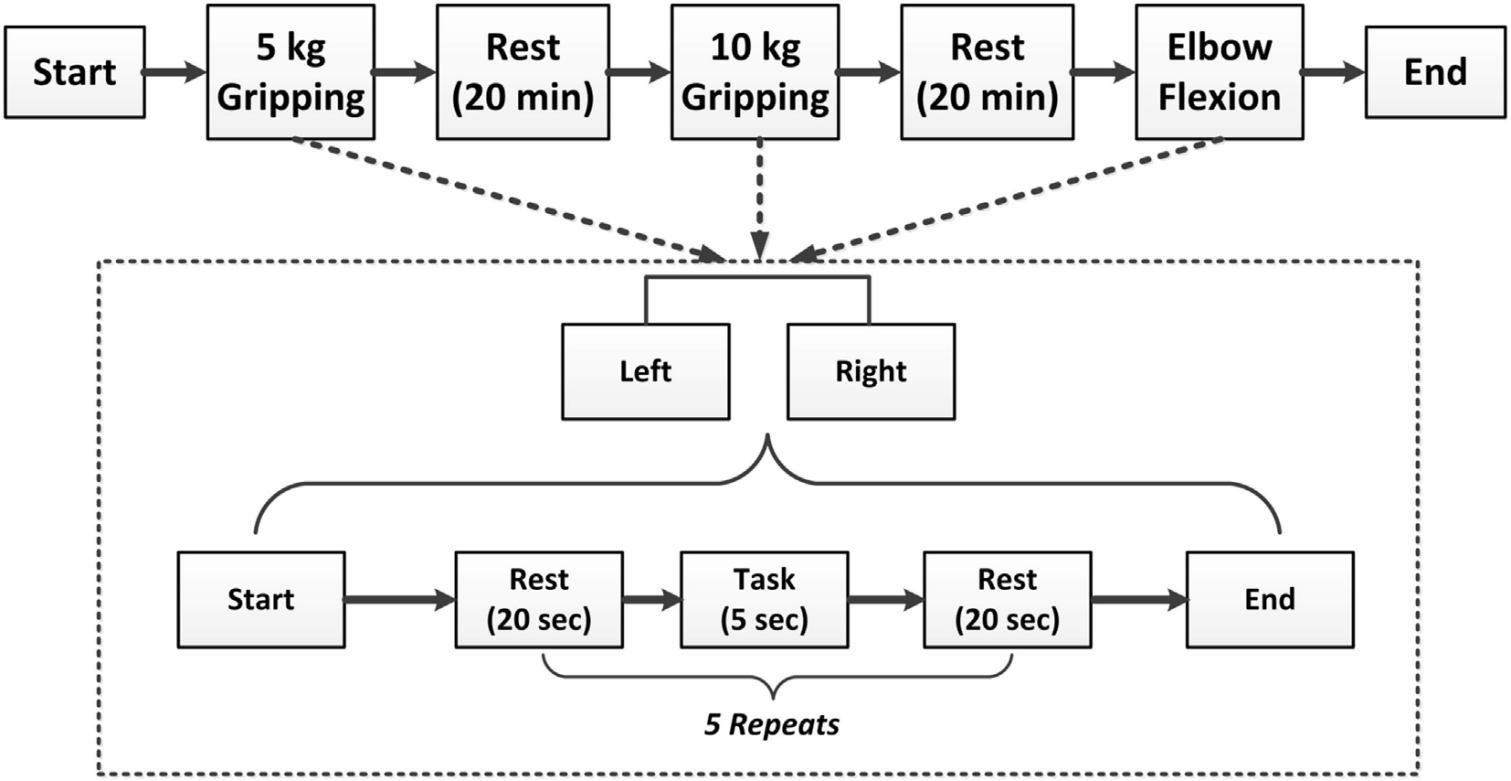
Illustration of the experimental paradigm.

To stabilize force outputs, a spring grip meter (EH101, Lynx Mall, China) was used in hand gripping tasks at 5 kg, 10 kg force levels, and elbow flexion task. The complete paradigm is illustrated in the Figure 1. All motor tasks for each subject started with a resting condition for 20 s, then subjects were asked to perform specific motor execution task for 5 s according to the instruction of a screen placed 1-m in front of their eyes, and then relaxed for 20 s. Each motor task contained five repeats, and all subjects performed each motor task using their left and right hand, respectively. After each motor task was completed, the subjects rested for 20 min before switched to next motor task to prevent muscle fatigue. Finally, the whole experiment ended up with 30 trials (2 hands × 3 tasks × 5 repeats) for each subject. As subjects S4 and S5 are severe stroke survivors, gripping tasks were only performed in the subject S4 at 5 and 10 kg force levels in both hands, and the 5 kg force level in both hands, and at 10 kg force level in the right hand in the subject S5. All other subjects successfully completed all experiments.

#### Data Collection

**Figure 2:**
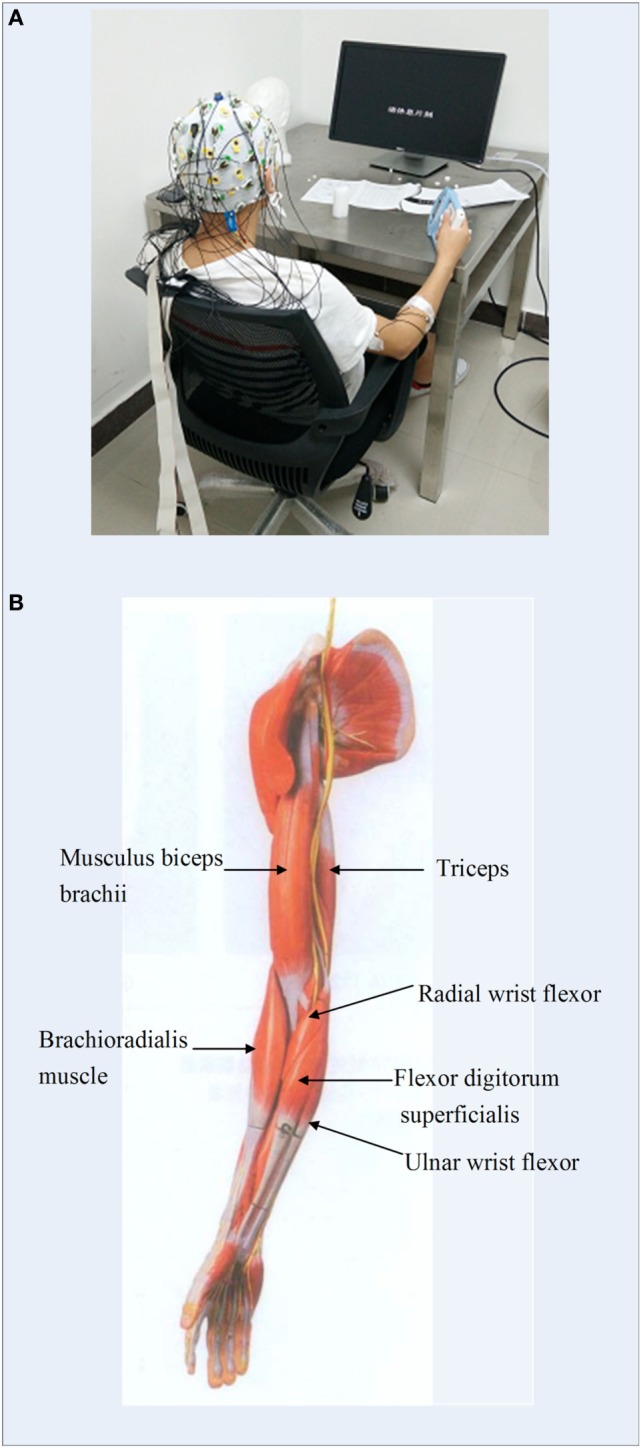
**(A)** Experimental environment of the electroencephalogram and electromyography (EMG) data measurement; **(B)** illustration of the locations of EMG electrodes on upper limb.

An EEG acquisition system (Brain Products GmbH, Germany) was utilized to collect 32-channel EEG signals from the whole head and 12-channel EMG signals from both sides of upper limbs (Figure 2A). EEG electrodes were placed on the scalp according to the international 10–20 standard system (FP1, FP2, F7, F8, F4, F3, FZ, FC5, FC1, FC2, FC6, T7, C3, CZ, C4, T8, CP5, CP1, CP2, CP6, TP9, P7, P3, PZ, P4, P8, TP10, PO9, O1, OZ, O2, PO10), and the binaural mastoid was used as reference electrodes. EMG signals were recorded from upper limb muscles including the flexor digitorum superficialis (FDS), brachioradialis muscle, radial wrist flexor, ulnar wrist flexor, musculus biceps brachii (MBB), and triceps (Figure 2B). The skin surface was carefully prepared and cleaned by alcohol before the electrodes were attached. The sampling frequency of EEG and EMG signals was set to 1,000 Hz.

#### EEG and EMG Signal Preprocessing

To study the coupling relationships between the EEG and the EMG signals associated with various motor tasks, the EEG signals of C3, C4, CP5, and CP6 channels, which covered the premotor cortex and the somatosensory cortex of the brain, and the EMG signals of the FDS and biceps muscle were selected for further analysis.

As the EEG and EMG signals are vulnerable and susceptible to noise such as powerline interference and baseline drift, such artifacts were subsequently removed by the EEG recording amplifiers and analysis software during data collection. The independent component analysis was employed to remove electrooculogram artifact, and the wavelet decomposition was employed to remove motion artifact (19) and improve the quality of EEG and EMG signals for further analysis.

### Variable-Scale Transfer Entropy Analysis

#### Time-Domain Analysis of EEG–EMG Signals

Symbolic transfer entropy analysis technique is an effective method to analyze the relationship between neural and muscular activity coupling. Symbolization (16–18), a technique processing coarse graining of the physiological signal before the calculation of TE, can capture large-scale dynamic characteristics of the signal and therefore reduce the effects of noise. For STE, the accuracy of symbolization affects the accuracy of the TE calculation and the dynamic coupling performance of the system. In particular, for traditional STE, fixed number of symbols is applied to symbolize the time sequence in advance. If the symbol sets is too large, data partitioning becomes smaller, which increases the computation cost and aggravates the noise. On the other hand, if the symbol set is too small, the data partition becomes thick, and the dynamic characteristics of signals are easily lost. To address the above shortcomings, a variable scale parameter symbolization method was proposed in this paper. The procedures of this proposed method is described as follow:

(1) Given a time series signal, the mean, maximum, and minimum values of the time series are first computed;
(2) A variable symbolic scale is set and denoted as piece, which segments the time series into pieces + 1 copies. The larger value of piece results in smaller segmentations and therefore more details of the signal can be retained;
(3) Then symbolize the time series. The segmentation fell into the smallest interval is assigned with the symbol $-\frac{\text{pieces}}{2}$, followed by $-\frac{\text{pieces}}{2}+0.5$, and so on. The largest symbol is $\frac{\text{pieces}}{2}$.

The specific function form is as shown in Eq. 1:

(1) $$S(i)=\left\{ \begin{array}{l} -\frac{\text{pieces}}{2}\;\begin{array}{cccc} \; & \; & \; & \min(x)\leq x(i)<\min(x)+\text{delta} \end{array}\\ -\frac{\text{pieces}}{2}+\begin{array}{ccc} \begin{array}{cc} 0.5 & \end{array}\;\min(x)+\text{delta}\leq x(i)<\min(x)+2*\text{delta} & \; & \; \end{array}\\ \begin{array}{cccc} \; & \; & \begin{array}{ccccc} \; & \; & \; & \; & \cdot \end{array} & \begin{array}{cccc} \; & \; & \; & \; \end{array} \end{array}\\ \begin{array}{cccc} \; & \begin{array}{ccccc} \; & \; & \; & \; & \; \end{array} & \cdot & \begin{array}{cccc} \; & \; & \; & \; \end{array} \end{array}\\ \begin{array}{ccc} \; & 0 & \;\min(x) \end{array}\begin{array}{ccc} \;+(\text{pieces}-1)*\text{delta}\leq x(i)<\min(x)+\text{pieces}*\text{delta}, & \; & \; \end{array}\\ \begin{array}{cccc} \; & \begin{array}{ccccc} \; & \; & \; & \; & \; \end{array} & \cdot & \begin{array}{cccc} \; & \; & \; & \; \end{array} \end{array}\\ \begin{array}{cccc} \; & \begin{array}{ccccc} \; & \; & \; & \; & \; \end{array} & \cdot & \begin{array}{cccc} \; & \; & \; & \; \end{array} \end{array}\\ \frac{\text{pieces}}{2}-0.5\;\begin{array}{cccc} \; & \;\;\max(x)-2*\text{delta}\leq x(i)<\max(x)-\text{delta} & \; & \; \end{array}\\ \frac{\text{pieces}}{2}\begin{array}{ccccc} \; & \; & \; & \; & \max(x)-\text{delta}\leq x(i)\leq \max(x) \end{array} \end{array}\right.$$

where *i* represents the length of the time series, *S*(*i*) represents the symbolized sequence, min(*x*) and max(*x*) represent the minimum and maximum values of the time series, delta represents the value of increasement per interval, which is $\frac{\text{max}(x)-\min(x)}{\text{pieces}+1}$.

TE is an indicator of the directional delivery of time series information, for instance, TE*_X_*_→_*_Y_* denotes the amount of information transferred from *X* to *Y*.

If given time series *X* = {*x*_1_, *x*_2_, …, *x_T_*} and *Y* = {*y*_1_, *y*_2_, …, *y_T_*}, where *T* is the length of the time series, *x*_1_, *y*_1_ are the first observation, and *x*_2_, *y*_2_ are the second observation of time series, respectively. The TE of *Y* to *X* is defined as TE*_Y_*_→_*_X_* shown in Eq. 2, and the TE of *X* to *Y* is defined as TE*_X_*_→_*_Y_* shown Eq. 3 (20–22):

(2) $${\text{TE}}_{Y\to X}=\sum_{x_{n+\tau },x_{n},y_{n}}p(x_{n+\tau },x_{n},y_{n})\log_{2}\left(\frac{p(x_{n+\tau },x_{n},y_{n})p(x_{n})}{p(x_{n},y_{n})p(x_{n+\tau },x_{n})}\right),$$

(3) $${\text{TE}}_{X\to Y}=\sum_{x_{n+\tau },x_{n},y_{n}}p(y_{n+\tau },x_{n},y_{n})\log_{2}\left(\frac{p(y_{n+\tau },x_{n},y_{n})p(y_{n})}{p(x_{n},y_{n})p(y_{n+\tau },y_{n})}\right),$$

where *n* is the discrete time index, τ is the predicted time, and *p*(⋅) represents the probability distribution.

Combining the variable scale parameter symbolization method and TE, a VS-STE approach was proposed in this paper to analyze the relationship between cerebral cortex and muscle electrical coupling and to explore the corticomuscular coupling. Generally, VS-STE is a method based on probability distribution and Shannon entropy to detect the asymmetry between time series, so as to obtain the causality between time series. In particular, the TE of EEG–EMG reflects the amount of information exchange between the cerebral cortex and motor neurons. Therefore, the TE of EEG to EMG represents the amount of information that the cerebral cortex transmits to the control muscle, and TE of EMG to EEG represents the amount of information that muscle cells feed back to the cerebral cortex.

#### Frequency-Domain Analysis of EEG–EMG Signals TE

After the pretreatment of Section “EEG and EMG Signal Preprocessing,” two sets of EEG and EMG time series signals were marked as *X* = {*x*_1_, *x*_2_, …, *x_M_*} and *Y* = {*y*_1_, *y*_2_, …, *y_M_*}, respectively. Then, the EEG and EMG signals, which ranged from 1 to 50 Hz, were filtered into 49 sub-band signals with a frequency interval of 1 Hz using a finite impulse response filter. Based on the definition of TE in Eqs 2 and 3, the TE of each sub-band of the EEG and EMG signals was expressed as TE*_X_*_→_*_Y_*(*f*) and TE*_Y_*_→_*_X_*(*f*), where *f* represents the sub-band frequency. In general, the greater the entropy, the larger the amount of information was transferred in this band.

#### Definition of Coupling Strength

To quantify the brain’s ability to control the arm and the arm’s response to the brain control command, a parameter named significant area was employed in this study to quantitatively describe the coupling strengths (CS) of EEG and EMG signals in different directions (16). Based on significant area, the CS from EEG to EMG is defined as CS*_X_*_→_*_Y_*, which shows the ability of the cerebral cortex to control the motor muscle, and CS*_Y_*_→_*_X_*, which indicates the response of the motor muscle to the control command, as shown in Eqs 4 and 5, respectively:

(4) $${\text{CS}}_{X\to Y}=\sum_{f}\Delta f\cdot {\text{TE}}_{X\to Y}(f),$$

(5) $${\text{CS}}_{Y\to X}=\sum_{f}\Delta f\cdot {\text{TE}}_{Y\to X}(f),$$

where Δ*f* represents the sub-band resolution, TE_X→Y_(*f*) and TE_Y→X_(*f*) represent the TE at the frequency *f* in different directions.

## Results

Theoretically the premotor area in the cerebral cortex is primarily activated when the subject performed motor task on the contralateral limb. The primary motor area (M1) is activated when the body maintains a movement, while the primary somatosensory area (S1) is activated when the sensation of the limb is received (10). Therefore, EEG signals from C3/C4 channel located in the primary motor zone, CP5/CP6 channel in the primary somatic sensory area, and EMG signals from the FDS, the bicipital muscle (MBB) channel were selected to study the TE in the article. For each 5-s motor task, the selected data length was *N* = 5,000.

### Determination of the Information Delay

**Figure 3:**
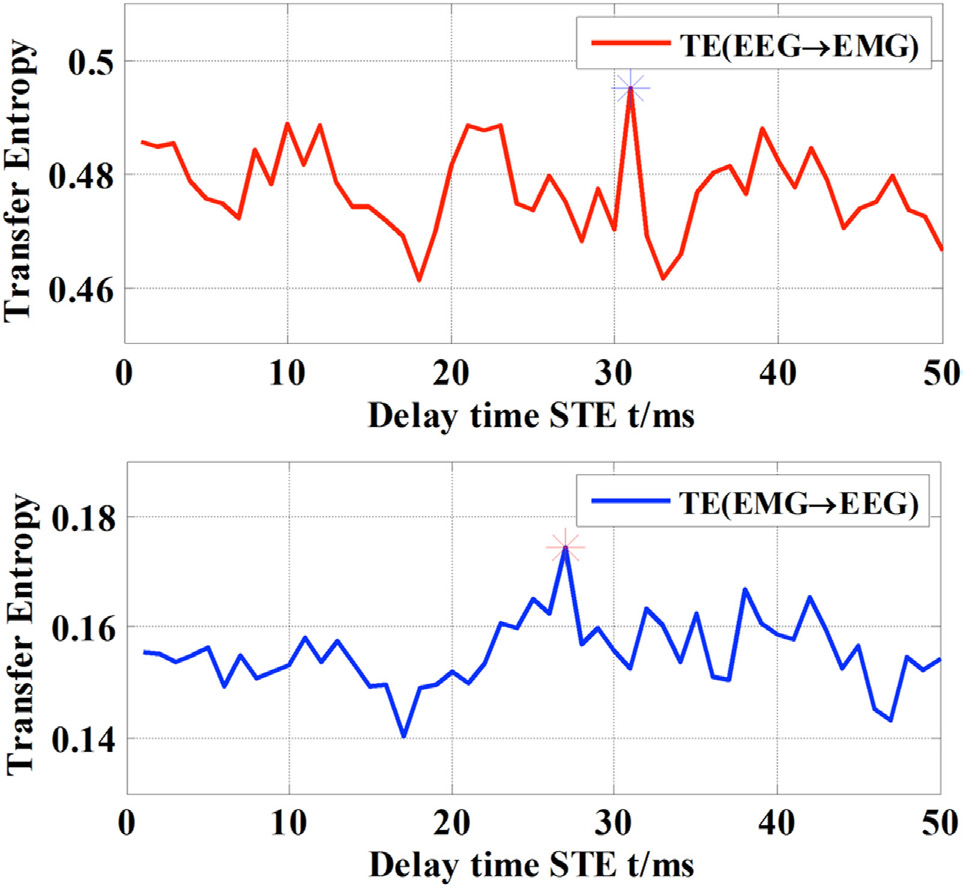
Different delay time of TE with respect to the direction of information flow.

There were certain delays in the flow of information in both directions between EEG and EMG (23). In particular, STE is believed to reach the peak between τ = 20 and 30 ms. Therefore, in this case, the STE of EEG-to-EMG and EMG-to-EEG were computed across all subjects by shifting the delay τ from 0 to 50 ms on the EEG signal of the C3 channel and the EMG signal of right hand’s FDS. Specifically, the delay of each individual subject was determined according to the optimal value of the STE (16). The STE of the subject S1 is shown in Figure 3 as an example, which shows that the delay of subject S1 is 31 ms from EEG to EMG and 27 ms from EMG to EEG. The summary of all subjects is shown in Table 2. It can be observed that delays of the descending (EEG to EMG) and ascending (EMG to EEG) pathways are different for individual subject, but generally concentrated around 20–30 ms, which is consistent with the results of a previous study (24).

**Table 2:** Delay time of all the subjects (ms).

	S1	S2	S3	S4	S5	S6	S7	S8	S9	S10	S11	S12
τ(EEG → EMG)/ms	31	27	25	29	31	21	25	23	26	19	24	27
τ(EMG → EEG)/ms	27	23	21	26	28	26	28	27	22	27	27	23

*EEG, electromyography; EMG, electroencephalogram*.

### Scale Parameter Selection of VS-STE

**Figure 4:**
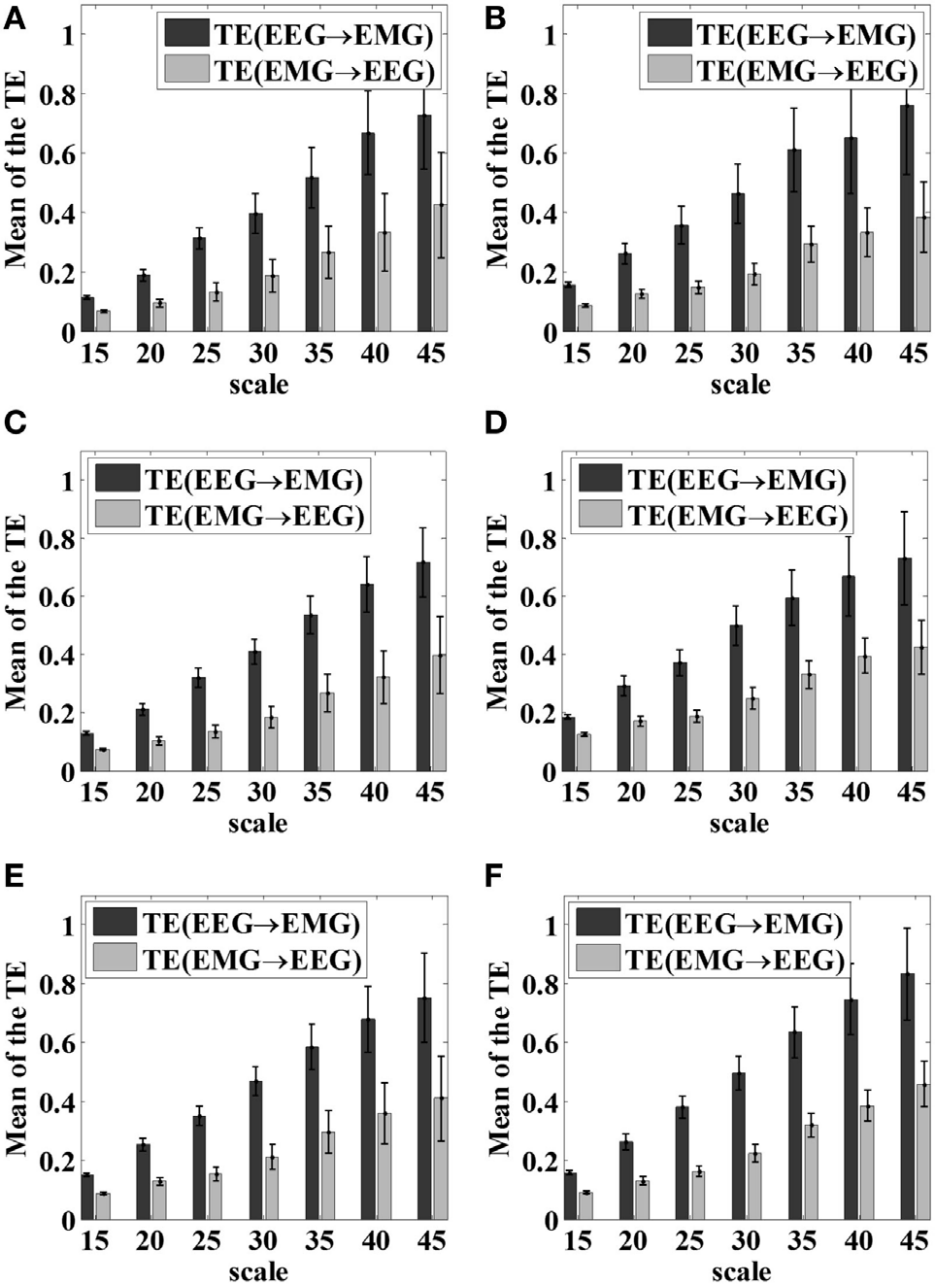
Mean and SD of the STE hand gripping task with respect to different scale parameters. **(A)** Left hand 5 kg gripping; **(B)** right hand 5 kg gripping; **(C)** left hand 10 kg gripping; **(D)** right hand 10 kg gripping; **(E)** left hand elbow bend; **(F)** right hand elbow bend.

As we introduced earlier, the scale parameter represents the degree of symbolization of the time series signal. If the number of symbols applied in the time sequence in STE is fixed beforehand, it is not flexible for further processing and the dynamic characteristics of the signals are easily lost. In this study, for each hand gripping task, EEG signals of C3/C4 channels and EMG signal of the FDS were first selected, then VS-STE method was used to analyze the coupling strength between EEG and EMG with respect to different scale parameters. For all subjects, the mean and SD of STE underwent 5 kg gripping, 10 kg gripping, and elbow flexion tasks in both hands after symbolization, are shown in Figure 4, respectively.

As shown in Figure 4, as the scale parameter *piece* increased, higher STE can be obtained from the symbolized time series, which indicated the loss of dynamic information was alleviated. However, it was also observed that the SD increased as the scale parameter increased, resulting in higher fluctuation of the STE. Therefore, it is necessary to comprehensively consider the mean and SD of STE to choose the scale parameter.

To select the appropriate scale parameters for symbolization, the objective function *G* was defined in Eq. 6 as follow in this article:

(6) $$\left\{ \begin{array}{l} G=a*M-b*S\\ a+b=1 \end{array}\right.,$$

where *M* and *S* denote the normalized mean and SD values of STE with respect to different scales, *a* and *b* are constants. Here, *a* and *b* are set to 0.5. The optimal scale parameter was then determined when the objective function reached its peak.

In this article, the scale parameter was set as 25 based on the Eq. 6, with which the time series was symbolized, then further coupling analysis of EEG–EMG signals was carried out.

### Analysis of Time-Domain STE in Subjects

**Figure 5:**
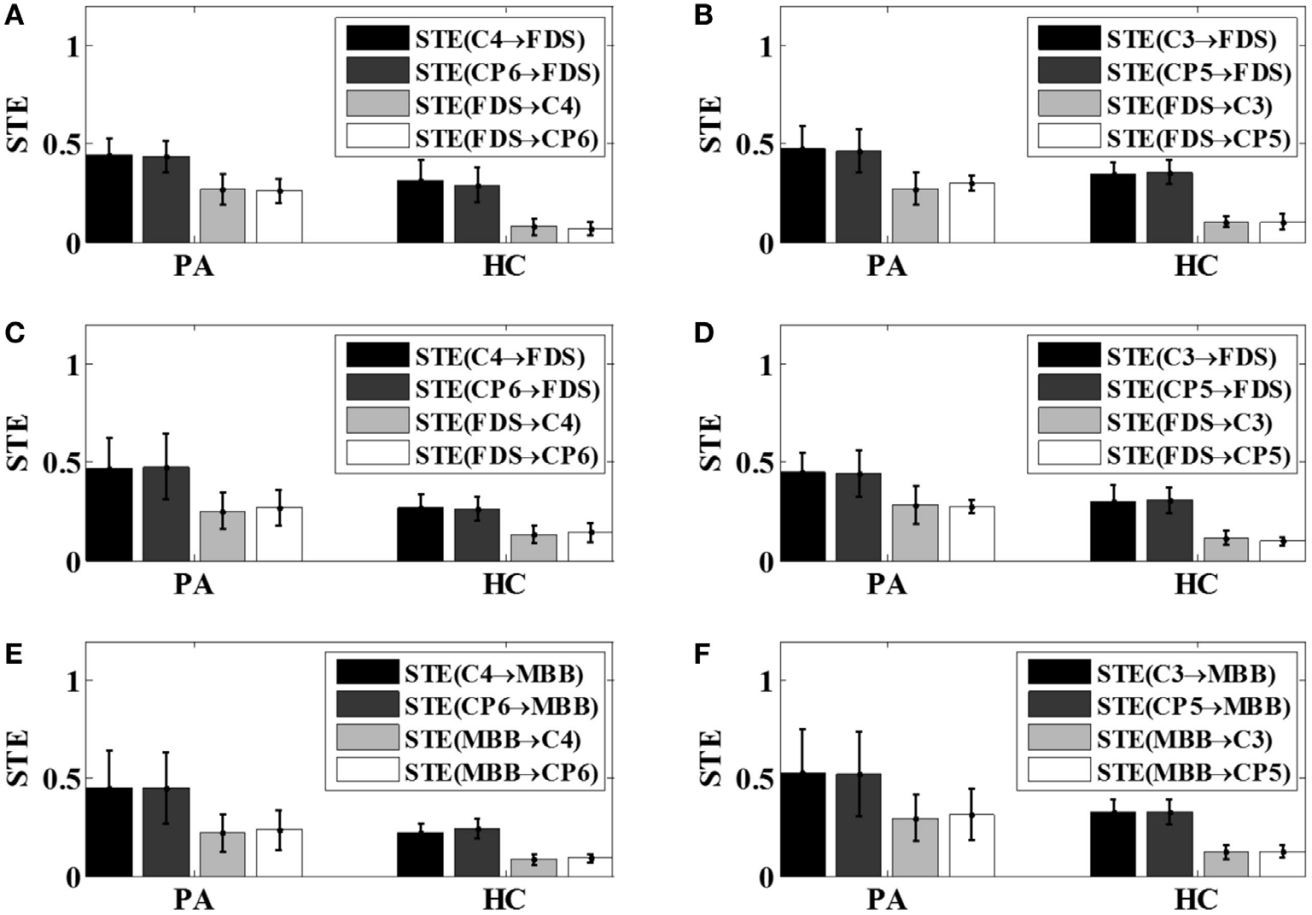
The bi-directional STEs between electroencephalogram and electromyography with respect to different tasks. **(A)** Left hand 5 kg gripping; **(B)** right hand 5 kg gripping; **(C)** left hand 10 kg gripping; **(D)** right hand 10 kg gripping; **(E)** left hand elbow flexion; **(F)** right hand elbow flexion. PA, patients; HC, healthy controls; STE, symbolic transfer entropy.

The bi-directional STE between EEG and EMG signals was computed using the pre-selected scale parameter for all motor tasks across all 12 subjects. The average bi-directional STEs between EEG and EMG signals of each group under different motor tasks were shown in Figure 5.

From Figure 5, it can be observed that for all motor tasks the mean value of STE from the EEG to EMG signals was greater than that from the EMG to EEG signals in the patient group as well as the control group. It can also be noticed that the mean value of the STE between the EEG and EMG signals in the patients tended to be higher than that of the healthy subjects, as demonstrated in Figure 5.

### Analysis of Frequency-Domain STE in Subjects

**Figure 6:**
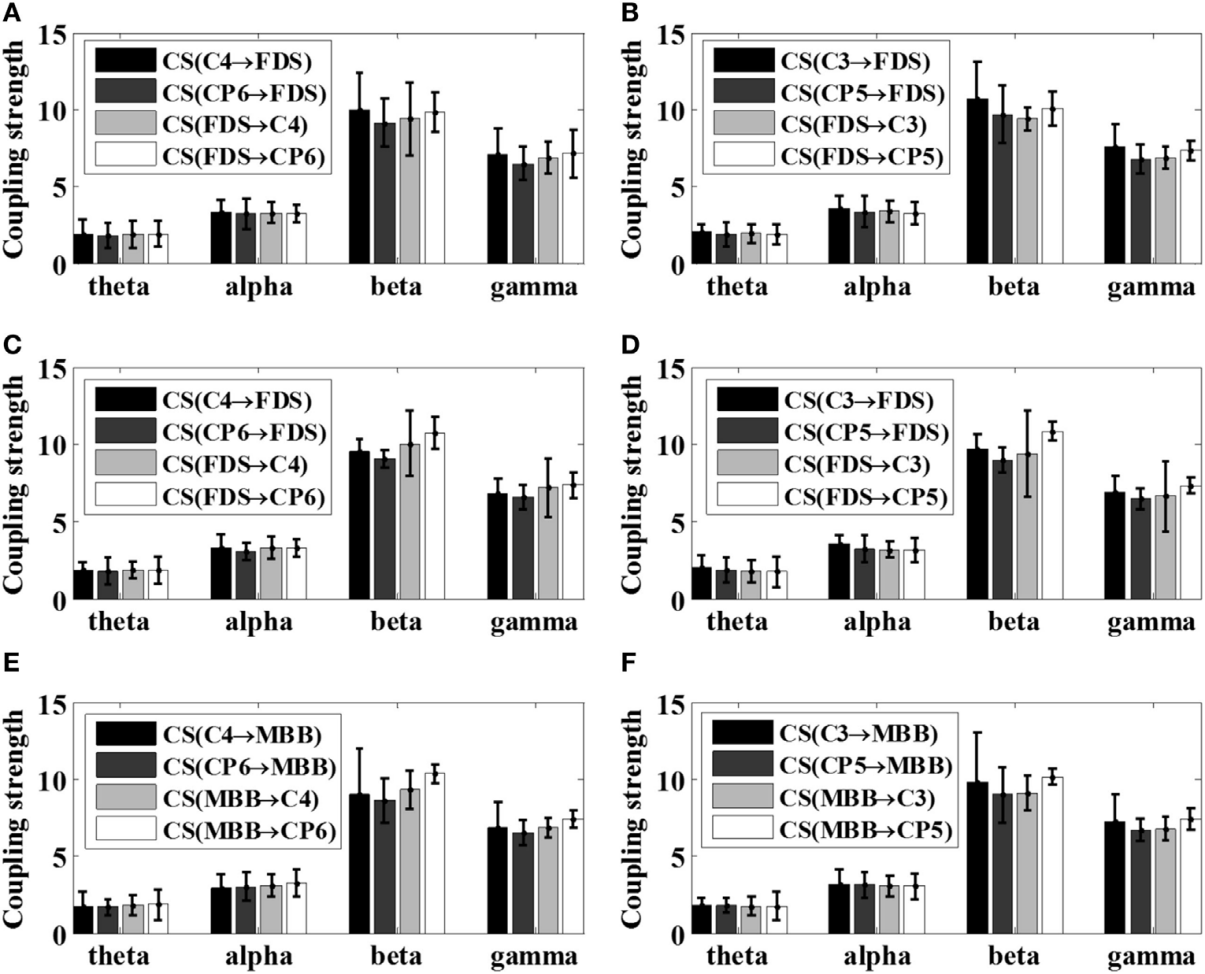
Summarized coupling strength (CS) of patient group (S1–S5) with respect to various motor tasks. **(A)** Left 5 kg; **(B)** right 5 kg; **(C)** left 10 kg; **(D)** right 10 kg; **(E)** left elbow flexion; **(F)** right elbow flexion.

**Figure 7:**
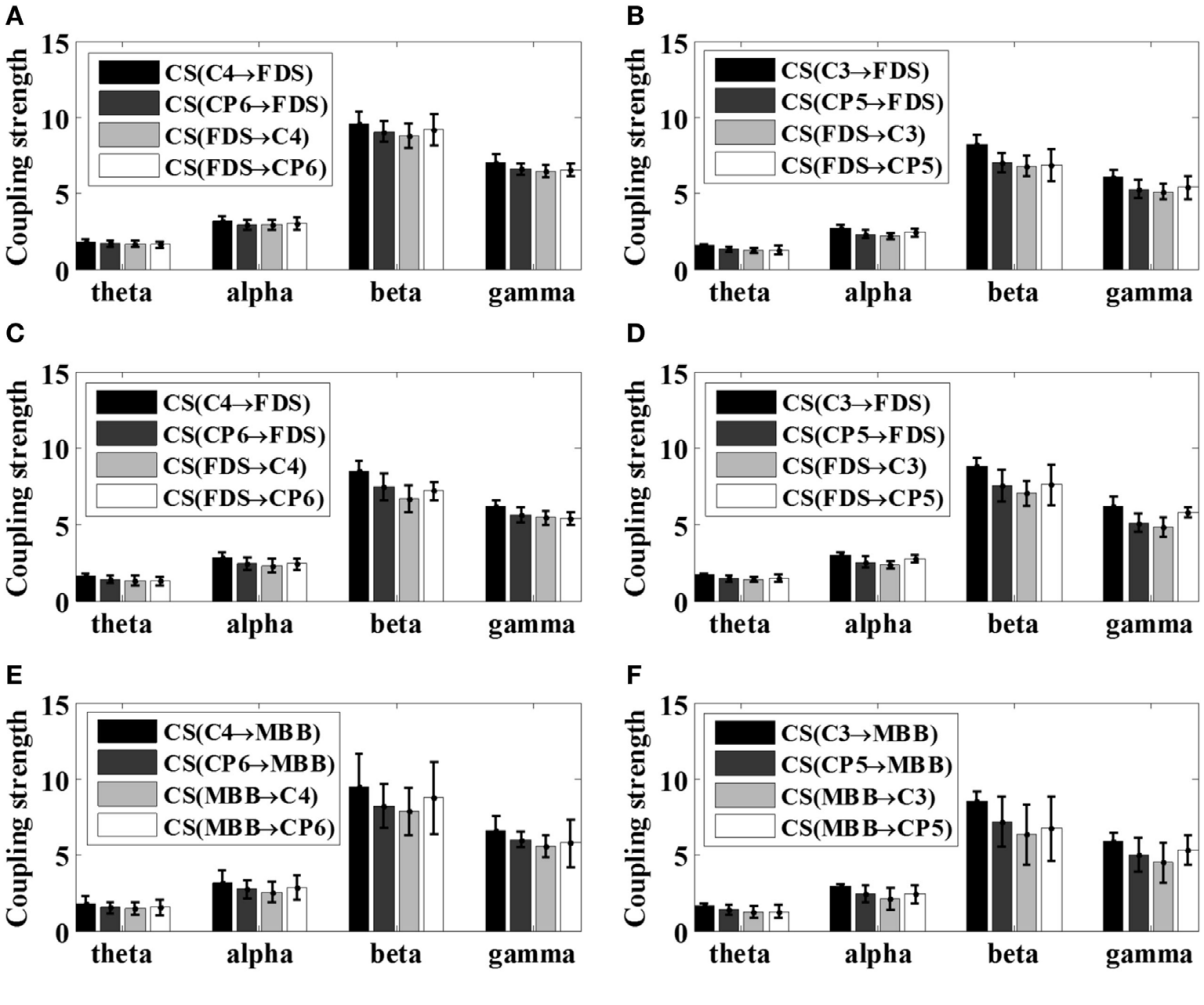
Mean coupling strength (CS) of healthy subjects (S6–S12) with respect to various motor tasks. **(A)** Left 5 kg; **(B)** right 5 kg; **(C)** left 10 kg; **(D)** right 10 kg; **(E)** left elbow flexion; **(F)** right elbow flexion.

Because different EEG rhythms may be involved in different ways during movement, the oscillatory responses of different frequency bands may be different with respect to various movements. Therefore, the STE between EEG and EMG signals were analyzed in multi-frequency bands for all subjects in this article. As reported in the previous study (16), significant area was employed to evaluate the coupling strength (CS) between EEG signals and EMG signals of specific frequency bands, including theta band (4–8 Hz, θ), alpha band (8–14 Hz, α), beta band (15–35 Hz, β), and gamma frequency band (35 Hz or more, γ). The results of the frequency-domain analysis for all the patients after stroke (S1–S5) and all healthy subjects were shown in Figures 6 and 7, respectively. The mean and SD of the coupling strength across all subjects were computed and summarized in Table 3.

**Table 3:** Comparison (mean ± SD) of the coupling strength across all subjects.

Tasks	Fre.	Group	Left hand		Right hand		Overall
			EEG > EMG	EMG > EEG	EEG > EMG	EMG > EEG	
5 kg	θ	PA	1.86 ± 0.43	1.89 ± 0.39	1.97 ± 0.48	1.90 ± 0.15	1.91 ± 0.05
		HC	1.74 ± 0.18	1.63 ± 0.20	1.42 ± 0.14	1.25 ± 0.19	1.51 ± 0.21
	α	PA	3.26 ± 0.75	3.28 ± 0.67	3.45 ± 0.79	3.31 ± 0.23	3.33 ± 0.09
		HC	3.05 ± 0.31	2.95 ± 0.36	2.48 ± 0.25	2.29 ± 0.24	2.69 ± 0.36
	β	PA	9.55 ± 1.39	9.60 ± 1.22	10.18 ± 1.58	9.74 ± 1.13	9.76 ± 0.29
		HC	9.31 ± 0.73	8.99 ± 0.93	7.63 ± 0.65	6.81 ± 0.87	8.19 ± 1.17
	γ	PA	6.79 ± 1.64	7.01 ± 1.54	7.16 ± 1.45	7.08 ± 0.83	7.01 ± 0.16
		HC	6.82 ± 0.44	6.49 ± 0.37	5.64 ± 0.55	5.26 ± 0.62	6.05 ± 0.72

10 kg	θ	PA	1.83 ± 0.52	1.87 ± 0.27	1.93 ± 0.39	1.79 ± 0.43	1.86 ± 0.06
		HC	1.50 ± 0.20	1.29 ± 0.29	1.58 ± 0.12	1.41 ± 0.20	1.45 ± 0.12
	α	PA	3.20 ± 0.85	3.30 ± 0.46	3.36 ± 0.58	3.16 ± 0.79	3.26 ± 0.09
		HC	2.62 ± 0.35	2.34 ± 0.38	2.76 ± 0.22	2.54 ± 0.24	2.57 ± 0.17
	β	PA	9.29 ± 0.51	10.39 ± 1.91	9.31 ± 1.06	10.10 ± 2.05	9.78 ± 0.56
		HC	7.95 ± 0.78	6.94 ± 0.74	8.18 ± 0.78	7.31 ± 1.07	7.59 ± 0.63
	γ	PA	6.72 ± 0.47	7.28 ± 1.31	6.71 ± 1.02	6.97 ± 1.71	6.92 ± 0.27
		HC	5.91 ± 0.42	5.39 ± 0.39	5.64 ± 0.63	5.30 ± 0.49	5.56 ± 0.44

Elbow bend	θ	PA	1.70 ± 0.60	1.82 ± 0.38	1.79 ± 0.62	1.75 ± 0.33	1.76 ± 0.05
		HC	1.68 ± 0.39	1.49 ± 0.47	1.53 ± 0.21	1.24 ± 0.42	1.48 ± 0.17
	α	PA	2.99 ± 0.58	3.17 ± 0.68	3.15 ± 1.07	3.06 ± 0.62	3.09 ± 0.08
		HC	2.97 ± 0.70	2.71 ± 0.76	2.67 ± 0.37	2.26 ± 0.65	2.65 ± 0.30
	β	PA	8.82 ± 2.44	9.83 ± 1.09	9.40 ± 1.97	9.64 ± 0.95	9.42 ± 0.44
		HC	8.87 ± 1.76	8.31 ± 1.98	7.87 ± 1.14	6.55 ± 2.04	7.90 ± 0.87
	γ	PA	6.67 ± 1.19	7.15 ± 0.79	6.69 ± 1.03	7.11 ± 0.76	6.98 ± 0.22
		HC	6.31 ± 0.74	5.67 ± 1.12	5.46 ± 0.86	4.92 ± 1.13	5.59 ± 0.43

*PA, patients; HC, healthy controls; EEG, electromyography; EMG, electroencephalogram*.

It is noticeable from Figures 6 and 7 that the coupling strength of EEG-to-EMG and EMG-to-EEG in the beta and gamma bands of all subjects were larger than theta and alpha bands during the execution of three motor tasks. In particular, for patients group, the mean value of the coupling strength in beta frequency band all exceeded 9.42 (9.65 ± 0.20), while the controls group showed similar results in beta frequency band 7.59 (7.89 ± 0.29). In addition, as shown in Figures 6 and 7 and Table 3, the results also demonstrated that in the beta band, the coupling strength from EEG to EMG was slightly higher than that from EMG to EEG band in control group, while for post-stroke patients the coupling strength from EEG to EMG appeared to be lower than that from EMG to EEG except 5 kg hand gripping in the right hand.

To better compare the difference of corticomuscular coupling strength between stroke patients and healthy subjects, according to the results in Table 3, the strength differences between the ascending neural pathway (EMG-to-EEG) and descending neural pathway (EEG-to-EMG) with respect to different motor tasks were evaluated by two sample *t* test using SPSS software (V22.0, IBM Corp., Armonk, NY, USA) within the β band. Results suggested that there is a significant difference (*p* < 0.05) in the coupling strength between patient group and control group, except for the 5 kg gripping task in right hand (*p* = 0.3272 > 0.05). As subject S4 and S5 were unable to complete the elbow flexion task due to severe stroke, the statistical test was not performed for elbow flexion task since the available samples in patient group was too small.

## Discussion and Conclusion

In this study, the corticomuscular coupling strength of both post-stroke patients (*n* = 5) and healthy volunteers (*n* = 7) were assessed under various motor tasks using the proposed VS-STE analysis method.

In time-domain, the VS-STE between EEG signals selected from the primary motor area and the somatosensory sensory area of the brain, and EMG signals was analyzed with respect to different motor tasks in all the five post-stroke patients and seven healthy controls. The results revealed that the STE from EEG to EMG signals was increased in patients after stroke during movements compared to healthy controls (Figure 5), which indicated that the amount of information transferred from the motor cortex to the muscles tended to increase in the post-stroke patients to complete the same movement. The reason may lie in the fact that more cerebral cortex areas, such as sensory motor cortex, auxiliary exercise area, pre-exercise area, and ipsilateral posterior parietal cortex area, were needed to be activated for the post-stroke patients to complete and maintain stable movements (23). In addition, the STEs from EMG to EEG in all motor tasks were also increased in patient group compared to those of control group, which may be caused by control disorder resulted from the damage of the motor function area and thereby prevent them activating the motoneuron and motor cortex exactly (25). The neural mechanism behind the appearance of abnormal coordination patterns during post-stroke recovery are largely unknown, but they are possibly related to a loss in cortical control and an increased usage of undamaged, indirect descending motor pathways *via* the brainstem (26).

In the frequency domain, the results showed in Figures 6 and 7 and Table 3 demonstrated that the corticomuscular coupling between the EEG and EMG signals was mainly reflected in the β bands and γ bands, indicating that the β and γ bands dominantly control the movement of the upper limb during exercise, which is consistent with the literature (27). One explanation lies in that the coupling of EEG and EMG occurs mainly in the beta band during the static force output, and then shifts to the high gamma band during the dynamic force output (11, 19). In addition, while the coupling strength from EEG to EMG in the beta band was slightly higher than that from EMG to EEG among healthy controls, post-stroke patients revealed lower coupling strength from EEG to EMG compared to the coupling strength from EMG to EEG except 5 kg hand gripping in the right hand. Studies have shown that the corticomuscular coupling in the beta band reflects the relative stable control state of the motor cortex, and the increased coupling strength from EMG to EEG in patients may indicate that intracranial hemorrhage may cause the neurons of the primary motor area unable to control steady movement, resulting an increase in the amount of information fed back to motor cortex to mobilize more neurons to work (27, 28). The above hypothesis is supported by the statistical analysis result of our proposed metric, which is the strength difference between the ascending neural pathway and descending neural pathway. Such result also demonstrated the capacity of the proposed VS-STE in evaluating the interaction between the cerebral cortex and the muscles. For the exception of 5 kg hand gripping in the right hand, which is regularly used and practiced in daily life, this may be attributed to the better recover progress in right hand among the patients during rehabilitation after the stroke (29).

One limitation of this preliminary study is that the results obtained by the proposed method cannot be effectively validated due to the small sample size of the subjects. However, we believed the current findings in this preliminary study do provide evidence and new insights to apply corticomuscular coupling assessment in the rehabilitative evaluation of motor function impairment after stroke. More subjects will be recruited in the future to further validate the proposed VS-STE analysis method.

## Ethics Statement

The study protocol was approved by the Institutional Review Board of Guangdong Provincial Work Injury Rehabilitation Hospital. Prior to the experiment, all the subjects were informed of the details of the experiments and signed the informed consent form.

## Author Contributions

YG contributed to the study design, data analysis, and paper writing; LR contributed to data analysis and paper writing; RL contributed to the study design, result interpretation, and paper writing; YZ contributed to the study design, experimental design, result interpretation, and paper writing.

## Conflict of Interest Statement

The authors declare that the research was conducted in the absence of any commercial or financial relationships that could be construed as a potential conflict of interest.
